# The Antimicrobial Peptide AMP-17 Derived from *Musca domestica* Inhibits Biofilm Formation and Eradicates Mature Biofilm in *Candida albicans*

**DOI:** 10.3390/antibiotics11111474

**Published:** 2022-10-25

**Authors:** Chaoqin Sun, Xinyu Zhao, Zhenglong Jiao, Jian Peng, Luoxiong Zhou, Longbing Yang, Mingjiao Huang, Chunren Tian, Guo Guo

**Affiliations:** 1 The Key and Characteristic Laboratory of Modern Pathogen Biology, School of Basic Medical Sciences, Guizhou Medical University, Guiyang 550025, China; 2 Key Laboratory of Environmental Pollution Monitoring and Disease Control, Ministry of Education, Guizhou Medical University, Guiyang 550025, China; 3 Center of Laboratory Medicine, the Affiliated Hospital of Guizhou Medical University, Guiyang 550025, China; 4 Translational Medicine Research Center, Guizhou Medical University, Guiyang 550025, China

**Keywords:** AMP-17, *C. albicans*, antibiofilm, adhesion, hyphae

## Abstract

The biofilm formation of *C. albicans* represents a major virulence factor during candidiasis. Biofilm-mediated drug resistance has necessitated the search for a new antifungal treatment strategy. In our previous study, a novel antimicrobial peptide named AMP-17 derived from *Musca domestica* was confirmed to have significant antifungal activity and suppress hyphal growth greatly in *C. albicans*. In the current work, we aimed to investigate the antibiofilm property of AMP-17 in *C. albicans* and explore the underlying mechanism. An antifungal susceptibility assay showed that AMP-17 exerted a strong inhibitory efficacy on both biofilm formation and preformed biofilms in *C. albicans*. Furthermore, AMP-17 was found to block the yeast-to-hypha transition and inhibit the adhesion of biofilm cells with a reduction in cellular surface hydrophobicity. A morphological analysis revealed that AMP-17 indeed suppressed typical biofilm formation and damaged the structures of the preformed biofilm. The RNA-seq showed that the MAPK pathway, biosynthesis of antibiotics, and essential components of the cell were mainly enriched in the biofilm-forming stage, while the citrate cycle (TCA cycle), phenylamine metabolism, and propanoate metabolism were enriched after the biofilm matured. Moreover, the co-expressed DEGs in the two pairwise comparisons highlighted the terms of transmembrane transporter activity, regulation of filamentation, and biofilm formation as important roles in the antibiofilm effect of AMP-17. Additionally, qRT-PCR confirmed that the level of the genes involved in cell adhesion, filamentous growth, MAPK, biofilm matrix, and cell dispersal was correspondingly altered after AMP-17 treatment. Overall, our findings reveal the underlying antibiofilm mechanisms of AMPs in *C. albicans*, providing an interesting perspective for the development of effective antifungal agents with antibiofilm efficacy in *Candida* spp.

## 1. Introduction

*Candida albicans* (*C. albicans*) is the most common human fungal pathogen and is responsible for notorious candidiasis ranging from superficial mucosal to life-threatening systemic infection. In regard to contemporary antifungals, *C. albicans* infections carry an alarming mortality in the United States, as high as 40%, in systemic candidiasis attributed to the incomplete eradication of the yeast and the emergence of resistant phenotypes [1]. The fungal pathogenicity is due to its virulence factors such as the ability to adhere to host tissues, the formation of hyphae, and the development of a complex and robust biofilm. *C. albicans* readily forms biofilms on the surface of the host or medical devices. It is known that biofilm formation poses a crucial cause for more drug resistance than its free yeast to conventional antifungal agents and that it is less affected by host immune responses. It has been estimated that microbes in biofilms can tolerate 100–1000 times higher concentrations of antibiotics than their counterpart planktonic microbes, making them harder to eradicate [2]. Even worse, mature biofilms continuously release *Candida* cells into the environment; these then colonize new sites, resulting in chronic infections [3]. Yeast and filamentous fungi biofilm-associated infections have been increasingly studied, and in 2021, this compelled the WHO to compile the first fungal priority pathogen list (FPPL) [4]. However, only a handful of antifungal drugs are currently approved for the treatment of fungal infections (e.g., azoles, polyenes, and echinocandins). Among these, the most broadly used azoles are ineffective against *C. albicans* biofilm due to the upregulation of efflux pumps in the biofilm, while amphotericin B, a representative of polyenes, failed to reach biofilms because of the enhanced extracellular matrix or beta-glucan synthesis during biofilm growth. Nevertheless, some *Candida* species with intrinsic resistance to fluconazole also have a decreased susceptibility to echinocandins, which are occasionally associated with serious bloodstream infections [5]. Furthermore, *Candida* spp. with an *fks* mutation can form resistance to echinocandins, reducing antibiofilm activity [6]. Consequently, the emergence of azole-resistant yeasts in many parts of the world draws grave concern. Even worse, the treatment of *C. albicans* biofilm-related infections is limited since the biofilm extracellular matrix acts as a physical barrier, thus preventing the diffusion of antimycotic drugs [7,8]. For these reasons, there is an urgent need to explore alternative therapeutic strategies for developing new antifungal drugs with efficient antibiofilm activity and non-toxicity, and novel drug targets are waiting to be discovered to solve the problem of *C. albicans* biofilm formation.

Recently, antimicrobial peptides (AMPs) have garnered attention for their broad-spectrum antimicrobial activities against bacteria, fungi, and some enveloped viruses [9,10,11,12], as well as their strong antibiofilm ability [13]. AMPs are natural, small, and highly conserved effector molecules that play a key role in innate immunity, with the antimicrobial mode of action mainly focused on the integrity of microbial wall cells by pore formation or related activities. AMPs are small polypeptide molecules generated by all living organisms to protect their host from pathogens, with properties of multiple cellular targets, low toxicity, and functional diversity. These characteristics enable the AMPs to exhibit potential ability as treatment options against organisms resistant to the conventional antifungal drugs and as a new treatment for biofilm-related infections [14,15]. So far, several natural peptides, including nisin, daptomycin, polymyxin B, and colistin, have been approved by the US Food and Drug Administration (FDA) for the clinical treatment of microbial infections [16].

In previous work, our research group discovered AMP-17, a novel antimicrobial peptide with a significant antifungal effect against *C. albicans*, *C. neoformans*, et al. [17,18], as well as exhibiting a low toxicity towards human erythrocytes and stable physicochemical characteristics [19]. Further studies showed that the antifungal activity of the peptide was closely associated with cell wall destruction, an increase in membrane permeability, filamentous growth delay, and the abnormal metabolism of macromolecules [17,20]. Given that filamentation as the structural basis of the robust biofilm acts an essential role in the *C. albicans* biofilm development, the inhibitory effect of AMP-17 on hyphae undoubtedly implies that it has potential antibiofilm ability. Therefore, it is necessary and valuable to investigate whether AMP-17 can inhibit *C. albicans* biofilm and classify the potential mode of the antibiofilm action.

According to the literature, the severity of *C. albican* biofilm has been linked to the increased cellular density associated with the biofilm community, *C. albicans* morphological changes such as yeast-hyphae transition, the protective effect of the extracellular matrix, and the production of drug efflux pumps [21]. Antifungal agents could play a crucial role in preventing and controlling biofilm-related infections if they target the aforementioned causes of biofilm formation. At present, the mechanisms of AMPs are referred to in the following ways: disruption of the cell signaling system, avoidance of excessive microbial reaction, suppression of binding protein transport, or degradation of the extracellular polymeric matrix [13]. Furthermore, the same AMPs can exert their biological activity in a corresponding way in different biofilm-forming stages [22]. In this work, we evaluate the antibiofilm activity of AMP-17 in the biofilm-forming and mature phase and summarize the specific antifungal characteristic of the peptide in different stages of the *C. albicans* biofilm.

Finally, to shed light on the antifungal mechanisms of AMP-17 against *C. albicans* biofilm and the putative targets, and considering that yeast germination and the filamentous growth in *C. albicans* play a significant role in biofilm formation, we performed morphological techniques such as SEM and CLSM to observe the structural features of *C. albicans* cells. RNA-seq was applied to study the gene expression profile of AMP-17-treated biofilms to find out the closely related response genes and signaling pathways, which could facilitate elucidating the molecular mechanisms of the peptide’s action on the *C. albicans* biofilm and open up new therapeutic alternatives for the treatment of *Candida*-related biofilm infection.

## 2. Results

### 2.1. Antifungal Effect of AMP-17 against C. albicans

In the current work, results showed the minimum inhibitory concentration (MIC) of AMP-17 against *C. albicans* SC5314 was 16 μg/mL, determined by the microdilution method to evaluate the antifungal activity of the peptide, which was consistent with our previous study [20]. In the control group, the MIC of fluconazole (FLC) against *C. albicans* 5314 and *C. parapsilosis* ATCC 22019 were 2 μg/mL and 1 μg/mL, respectively.

### 2.2. AMP-17 Inhibits C. albicans Biofilms In Vitro

**Figure 1:**
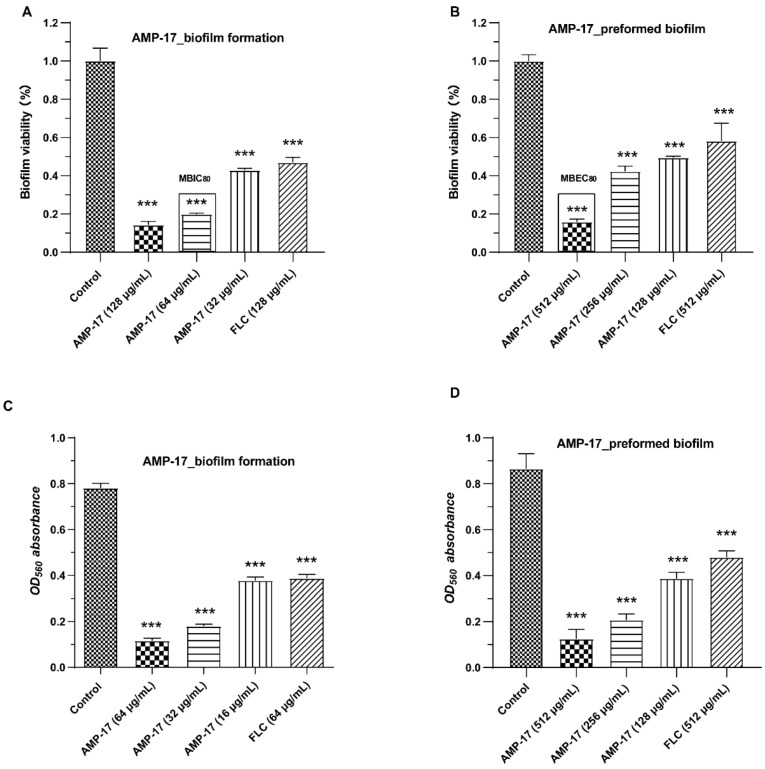
Effect of AMP-17 on biofilm formation and mature biofilm in *C. albicans*. *C. alibicans* biofilms were grown in RPMI 1640—10% FBS at 37 °C. Experiments related to biofilm formation were performed after *C.alibicans* was co-incubated with or without AMP-17 for 12 h. The antibiofilm effect of AMP-17 on mature biofilm was not evaluated until the *C. albicans* biofilm grew to maturity after incubation for 24 h. The preformed biofilm was then treated for another 12 h in RPMI 1640 with or without AMP-17. (**A**) The biofilm metabolic activity in the early biofilm-forming stage as determined by the XTT assay. (**B**) The biofilm metabolic activity after the biofilm matured as determined by the XTT assay. The biofilm viability was calculated using Equation (1) (see Methods) and normalized to the control group. (**C**) The OD560 nm absorbance of the destained biofilm solution was measured by the crystal violet (CV) method in the biofilm-forming stage. (**D**) The OD560 nm absorbance of the destained biofilm solution was measured by the CV method after the biofilm matured. Experiments were repeated three times; data points represent the means of three independent experiments, and error bars denote standard deviations. *** *p* < 0.001 were obtained for control versus treated samples.

Both a 2,3-bis-(2-methoxy-4-nitro-5-sulfophenyl)-2H-tetrazolium-5-carboxanilide salt (XTT) reduction assay and the Crystal Violet (CV) method revealed that AMP-17 inhibited biofilm formation and effectively eradicated preformed biofilm in *C. albicans* in a dose-dependent manner (Figure 1). The XTT assays found, first, that levels of suppression of biofilm formation reached 57%, 80%, and 86%, in response to AMP-17 at the concentrations of 32 μg/mL, 64 μg/mL, and 128 μg/mL, respectively (Figure 1A). While 128 μg/mL FLC only reduced the biofilm by 53%, it had comparable antibiofilm activity to 32 μg/mL AMP-17 during the biofilm-forming stage. Second, the inhibitory assay of mature biofilm revealed that more than 80% of preformed biofilms were removed when treated with 512 μg/mL AMP-17 (Figure 1B). These effects were also seen in a CV staining experiment. In the early and developmental stages of biofilm formation, with the increasing dose of AMP-17, the antibiofilm effect against early biofilm formation was evident. AMP-17 at concentrations of 16 μg/mL, 32 μg/mL, and 64 μg/mL reduced biofilm formation by approximately 51%, 77%, and 86%, respectively, whereas less than 50% of biofilms were inhibited in response to 64 μg/mL FLC (Figure 1C). At the mature biofilm stage (24 h), the eradication effect of AMP-17 against preformed biofilm was also found to be stronger than that of FLC. For example, 512 μg/mL AMP-17 can remove 86% of mature biofilm, and FLC at the same concentration of 512 μg/mL resulted in a reduction in biofilm activity of merely 44% (Figure 1D). These results indicate that AMP-17 significantly inhibited early biofilm formation and mature biofilm in *C. albicans*, with the Minimum Biofilm Inhibitory Concentration (MBIC_80_) and Minimum Biofilm Eradication Concentration (MBEC_80_) being 64 μg/mL 512 μg/mL, respectively.

**Figure 2:**
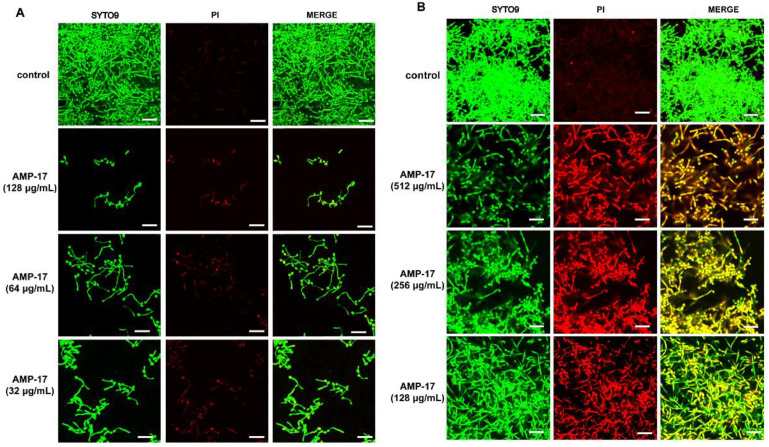
The effect of AMP-17 against biofilm formation and mature biofilm was shown in CLSM. Biofilms were stained with SYTO9 and PI, and images were captured using a confocal laser scanning microscope. Live cells were stained in green and dead cells in red. (**A**) The effect of AMP-17 on biofilm formation *C. albicans* cells (10^6^ CFU/mL) incubated on plastic coverslips at 37 °C for 12 h in the presence or absence of AMP-17 at concentrations of 32 μg/mL, 64 μg/mL, and 128 μg/mL. The AMP-17-treated *C. albicans* biofilms exhibited a few cells adhered and no biofilm structure was found. (**B**) The effect of AMP-17 against mature biofilm. After the *C. albicans* biofilm was incubated at 37 °C for 24 h to mature, it was treated with different concentrations of AMP-17 (128 μg/mL, 256 μg/mL, and 512 μg/mL). Preformed biofilms exposed to AMP-17 exhibited a high dead cell rate in the biofilms. SYTO9 and PI were used to distinguish viable and dead cells. Bar, 20 μm.

**Figure 3:**
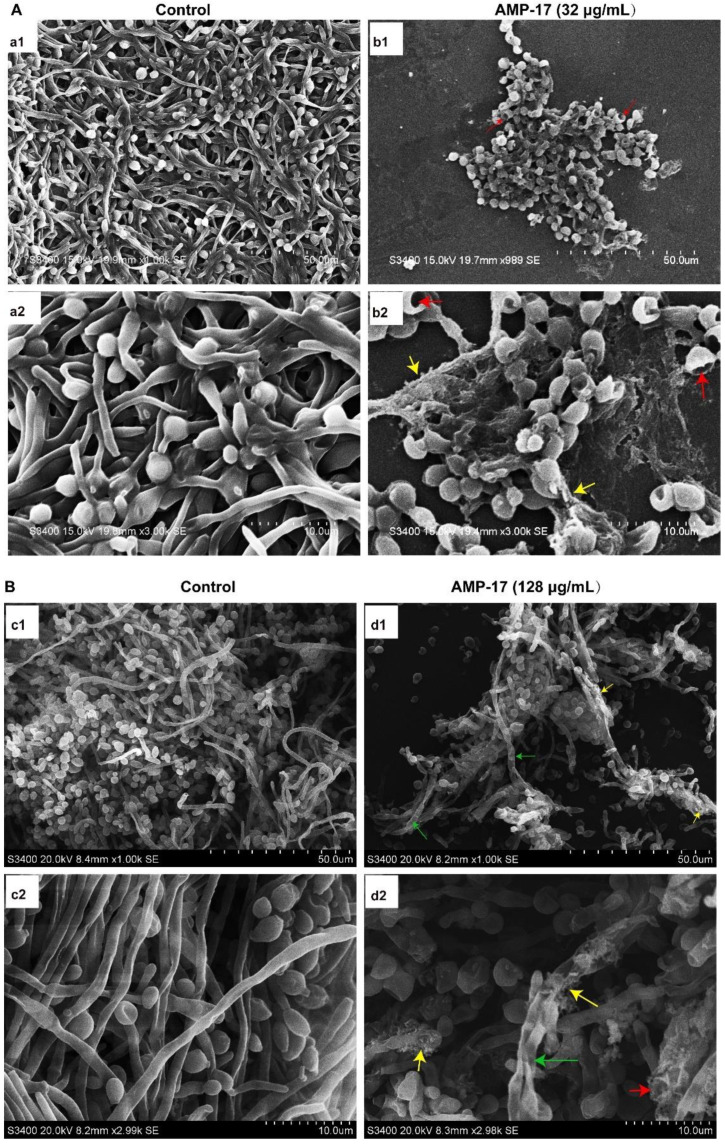
SEM images showed AMP-17-inhibited biofilm formation and mature biofilms in *C. albicans*. (**A**) Inhibitory effect of 32 μg/mL AMP-17 on biofilm formation. Biofilms were inhibited when exposed to AMP-17 with the appearance of broken yeast cells (red arrows), rough surfaces (yellow arrows), and the avoidance of biofilm architecture. (**B**) Anti-preformed biofilm effect of 128 μg/mL AMP-17. Biofilms appeared with abnormal structures including broken yeast cells (red arrows), fractured hyphae (green arrows), and a loosening extracellular matrix (yellow arrows). The biofilms were visualized under magnification of 1000× (**a1**, **b1**, **c1** and **d1**) and 3000× (**a2**, **b2**, **c2** and **d2**).

Confocal laser scanning microscopy (CLSM) and scanning electron microscopy (SEM) assays further confirmed the antibiofilm effect of AMP-17 against biofilm formation and preformed biofilm. *C. albicans* biofilms in the control group developed into a crisscrossing network of three dimensions (3D), with long and strong filaments expanding robustly as shown in Figure 2 and Figure 3. Biofilms are highly structured microbial communities that are wrapped in an extracellular polymer matrix [23]. Morphological images indeed found dense microbial populations composed of yeast cells and mycelia in the control group.

In the biofilm-forming stage (12 h), biofilm formation was reduced in a dose-dependent manner (Figure 2A) and nearly no biofilm was detected in the presence of AMP-17 at concentrations ranging from 32 μg/mL to 128 μg/mL. When exposed to AMP-17, the numbers of both planktonic cells and hyphae were lower than the numbers in the drug-free control. Upon treatment with AMP-17 at 32 μg/mL, the viability of the hyphae was decreased, as visualized by SYTO9 labeling living cells, analyzed by CLSM. The higher the concentrations of AMP-17 were, the less biofilm formed, and the smaller the number of living cells and the shorter the length of the *C. albicans* filament (Figure 2A). SEM assays showed that the density of biofilm cells was reduced, and large amounts of cells were restricted at the budding yeast stage. At 32 μg/mL AMP-17, no long hyphae were seen, yeast cells were broken (red arrows) and the extracellular matrix turned loose and coarse (yellow arrows), as seen in Figure 3A(b1,b2).

After the biofilm matured, the preformed *C. albicans* biofilms exposed to AMP-17 demonstrated a significant reduction or damaged structure, analyzed by CLSM and SEM. Preformed, mature biofilms exhibited a more dense and robust structure with long and strong filaments crisscrossing in the 3D network, as shown in the control group. In contrast, the AMP-17-treated-preformed biofilms were strikingly different from the drug-free samples. When exposed to AMP-17, the preformed biofilm exhibited large amounts of dead cells that were labeled by Propidium Iodide (PI) and emitted red fluorescence. The higher the AMP-17 treatment, the more dead cells were found in the preformed biofilms (Figure 2B), which corroborated the reductive metabolism of AMP-17-treated preformed biofilms in XTT or CV assays. In addition, this disruptive effect of AMP-17 against mature biofilms was also displayed in the SEM images (Figure 3B). The preformed biofilms treated with 128 μg/mL AMP-17 presented abnormal biofilm architecture such as broken yeast cells (red arrows), rough extracellular matrix (yellow arrows), and fractured hyphae (green arrows), as shown in Figure 3B(d1,d2). Therefore, the findings exhibited structurally scant, amorphous biofilms that were haphazardly and unevenly arranged when the preformed biofilm was treated with AMP-17.

### 2.3. In Planktonic C. albicans, AMP-17 Reduces Hydrophobicity and Inhibits Adhesion

**Figure 4:**
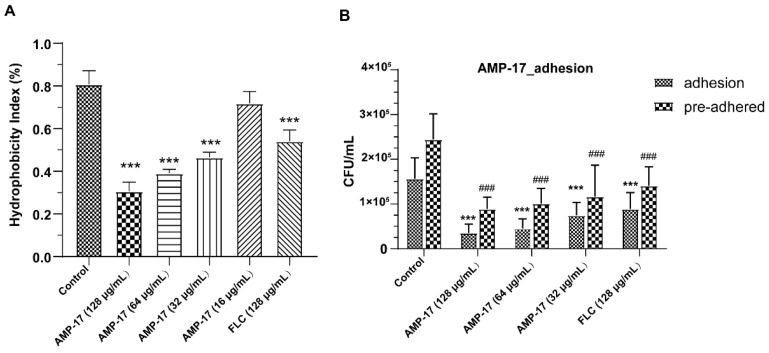
The effect of AMP-17 on the hydrophobicity and adhesion of *C. albicans*. (**A**) Hydrophobicity index (%) of *C. albicans* SC5314 after exposure to various AMP-17 concentrations. (**B**) The number of *C. albicans* cells that adhered on a 24-well polyethylene plate after 90 min of exposure to AMP-17. Experiments were repeated three times on three separate occasions and the results are displayed as the mean of each individual experiment, with the calculated standard deviation shown as error bars. *** *p* < 0.001 or ### *p* < 0.001 were obtained for control versus corresponding treated samples.

*C. albicans* must go through two essential stages during biofilm formation: adhesion and morphological transition. The first step is positively related to the cell surface hydrophobicity of *C. albicans* [24]. We found the hydrophobicity index (HI) of the control group to be 0.81, and that the HI underwent a reduction in a dose-dependent manner in response to AMP-17 (Figure 4A). Compared to the control group, the HI of AMP-17 at the concentrations of 128 μg/mL, 64 μg/mL, and 32 μg/mL decreased by 0.50, 0.42, and 0.34, respectively, demonstrating the weaker hydrophobicity of the AMP-17-treated *C. albicans* cell surface than FLC. In addition, AMP-17 indeed inhibited *C. albicans* cells adhering to the surface of the microplate and reduced the counts of the adhered cells in a dose-dependent manner (Figure 4B), corresponding with the HI results. Compared to the control, 32 μg/mL AMP-17 decreased nearly 50% of cells adhering or adhered on the surface of the plate, while FLC at a concentration of 128 μg/mL led to a similar effect. Moreover, *C. albicans* cells reduced the rate of adhering by about 71% and 77% when exposed to AMP-17 of 64 μg/mL and 128 μg/mL, respectively. For inhibitory effects of the peptide against preformed adhered *C. albicans*, AMP-17 at concentrations of 64 μg/mL and 128 μg/mL eliminated the adhered cells by 59% and 64%, respectively. These data suggest that AMP-17 could inhibit the hydrophobicity and adhesion of *C. albicans*, causing initial biofilm formation to likely be suppressed.

### 2.4. AMP-17 Prevents Yeast-to-Hyphae Transition

**Figure 5:**
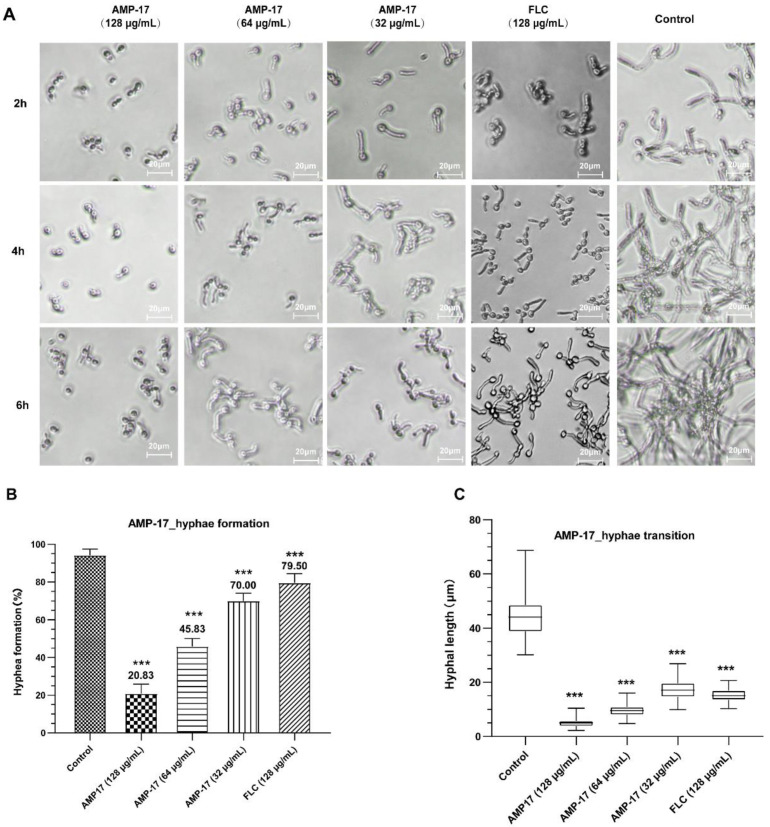
The effect of AMP-17 on the yeast-to-hypha transition and filamentous growth in *C. albicans*. (**A**) The effects of AMP-17 on yeast-to-hypha transition in RPMI 1640 medium. AMP-17 was diluted in RPMI 1640-FBS medium to final concentrations of 32 µg/mL, 64 µg/mL, and 128µg/mL, and FLC to the concentration of 128 µg/mL as a positive control. Bar, 20 µm. (**B**) The hyphae formation rate of *C. albicans* cells exposed to different concentrations of AMP-17 in RPMI medium for 2 h. (**C**) The hyphal length of *C. albicans* after treatment with different concentrations of AMP-17 in RPMI medium for 2 h, measured using Image J. Error bars represent the standard deviation of three independent experiments. *** *p* < 0.001 compared to the control group.

The yeast-to-hypha transition of *C. albicans* has been considered a crucial virulence factor that plays an important role in biofilm development and maintenance. In the present study, *C. albicans* hyphae induced by RPMI 1640-FBS are displayed in Figure 5. As shown, the length and density of *C. albicans* hyphae increased along with the incubation time (Figure 5A), and *C. albicans* generated long and crisscrossing filamentation to form a network 6 h after incubation in the control group. Moreover, radical colonies of untreated *C.albican* cells with feathery edges on solid Spider medium were observed (Figure S1), indicative of true hyphal growth. When increasing AMP-17 dosages, there was a dose-dependent inhibitory effect of the peptide on hyphal formation. Furthermore, 32 μg/mL AMP-17 was able to inhibit about 30% of *C. albicans* yeast budding (Figure 5B), shorten the length of hypha by more than two times (Figure 5C), and reduce the yeast cell aggregates after incubation for 2 h, compared with the control group. Additionally, at higher AMP-17 concentrations, cell densities were significantly reduced and filamentation was greatly restrained. Whereas, at a concentration of 128 μg/mL, FLC inhibited filamentation in *C. albicans* only slightly, less than the effect of 32 μg/mL AMP-17 during the observed period. Accordingly, cells grown on solid Spider medium demonstrated smooth-edged colonies under treatment with 8 μg/mL of AMP-17, suggesting only yeast growth inside the colony (Figure S1). Therefore, AMP-17 inhibited the *C. albicans* yeast-to-hypha transition and filamentous growth in a dose-dependent manner in both liquid and solid hypha-inducing media.

### 2.5. AMP-17 Affects the Transcriptome of C. albicans Biofilm

#### 2.5.1. Transcriptional Profiling of *C. albicans* Biofilm by RNA-Seq

To better understand the underlying molecular mechanism of AMP-17 on *C. albicans* biofilm, transcriptional profiles of early biofilm and mature biofilm treated with AMP-17 were performed by RNA-seq. An average of 6.36 Gb of database for each sample was obtained after trimming the unqualified read. The summary characteristics of the RNA-seq data are provided in Table S3. The global transcriptional profile of *C. albicans* biofilms was conducted using criteria log_2_(fold change) > 1, adjust *p* < 0.05, and the overall distribution of differentially expressed genes (DEGs), visualized using a volcano map.

**Figure 6:**
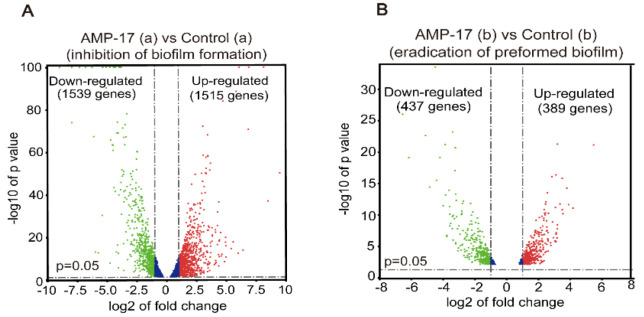
The effect of AMP-17 on the transcriptional profiling of biofilms in *C. albicans*. (**A**) The volcanic map analysis of DEGs in *C. albicans* biofilm formation after 12 h of treatment with 32 μg/mL AMP-17. (**B**) The volcanic map analysis of DEGs in preformed *C. albicans* biofilm after 12 h of treatment with 128 μg/mL AMP-17. “a” in the above figure indicates early forming biofilm (12 h), while “b” indicates preformed biofilm.

The transcriptome of *C. albicans* biofilms in the two pairwise comparisons both changed significantly after AMP-17 treatment. First, in the biofilm-forming stage, a total of 3054 DEGs, including 1539 upregulated and 1515 downregulated genes, were identified in the 32 μg/mL AMP-17 treated samples, compared with the control group, as shown in the volcano chart Figure 6A. Second, for mature biofilms, a total of 826 DEGs, including 437 upregulated and 389 downregulated genes, were found with 128 μg/mL AMP-17 treatment (Figure 6B). These DEGs are related to adhesion, filamentous growth, biofilm formation, metabolism, transport, membrane composition, etc. (Tables S1, S4 and S5). In the Venn Diagram, there are 172 upregulated DEGs and 153 downregulated DEGs in both the biofilm-forming and mature biofilm period after AMP-17 treatment (Figure S2). These DEGs that are identified in both states might play an important role in the antibiofilm efficacy of AMP-17 during the whole *C. albicans* biofilm process and deserve further analysis and study.

#### 2.5.2. Functional Annotation and Classification

**Figure 7:**
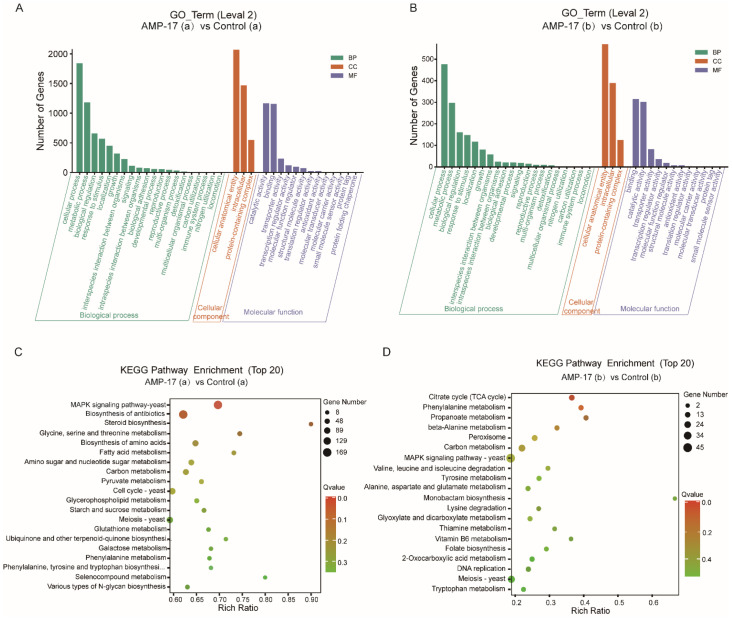
The functional annotation and classification of DEGs. (**A**,**B**) The GO classification of DEGs in the biofilm formation of *C. albicans* after 32 μg/mL AMP-17 for 12 h (**A**) or preformed biofilm of *C. albicans* after 128 μg/mL AMP-17 for 12 h (**B**). (**C**,**D**) The scatter plot of KEGG enrichment of DEGs in the biofilm formation of *C. albicans* after 32 μg/mL AMP-17 (**C**) or preformed biofilm of *C. albicans* after 128 μg/mL AMP-17 (**D**). “a” in the above figure indicates early forming biofilm (12 h), while “b” indicates preformed biofilm.

The possible functions of the identified DEGs were predicted using the gene function classification system Gene Ontology (GO), which has been segmented into three ontologies including molecular function (MF), cellular components (CC), and biological process (BP). A total of 2548 annotated DEGs were categorized into 36 GO functional groups in the biofilm-forming phase (Figure 7A) and 697 annotated genes were categorized into 35 GO functional groups in the mature biofilm phase (Figure 7B). Most of the genes were enriched in the biological process category, followed by the molecular function and cellular component categories, in both pairwise comparisons. Furthermore, most GO terms on level (2) clustered in the three categories are the same in the two pairwise, for example, the terms “cell process”, “metabolic process”, “biological regulation”, “growth”, and “biological adhesion” appeared to be clustered in BP category, the same three terms are clustered in the CC category, and the first three terms in the MF category, including “binding”, “catalytic activity”, and “transporter activity” all occurring in the two pairwise comparisons (Figure 7A,B). As a result, the GO analysis suggests that AMP-17 interfered with a large number of the same functional molecules involved in many similar biological processes such as adhesion, growth, catalysis, metabolism, transport, transcription, and so on, even in the different biofilm developmental stages.

For KEGG analysis, a total of 1326 and 368 DEGs after AMP-17 treatment were involved in various pathways, in the early biofilm-forming and mature biofilm stages, respectively (Figure 7C,D). Although there are many similar terms clustered in the same category in the two pairwise comparisons, the enriched pathways in the two pairwise comparisons are distinctively different. The DEGs of 32 μg/mL AMP-17-treated forming biofilms revealed the main enriched pathways were the MAPK signaling pathway-yeast, biosynthesis of antibiotics, steroid biosynthesis, glycine, serine, and threonine metabolism, and biosynthesis of amino acids (Figure 7C). It is indicated that AMP-17 suppressed biofilm formation, probably through the abovementioned pathways. It has been well-known that hyphal formation and biofilm development in *C. albicans* are regulated by the Ras-MARK pathway [25,26]. While in the mature biofilm state, the top five enriched pathways were the citrate cycle (TCA cycle), phenylamine metabolism, propanoate metabolism, beta-Alanine metabolisms, and peroxisome (Figure 7D). To the best of our knowledge, once the *C. albicans* biofilm matured, a mass of extracellular polymeric matrix consisting of proteins, polysaccharides, and DNA was produced, contributing to the difficult removal of biofilm and invasive and obstinate infection [27]. Through KEGG enrichment analysis, the DEGs of 128 μg/mL AMP-17-treated preformed biofilm were mostly found to be enriched in functions related to metabolisms, such as amino acids, carbohydrates, lipids, or glycans. These processes are not only needed to maintain growth but are likely to take part in the degeneration of the extracellular polymeric matrix. Meanwhile, we found a lot of DEGs encoding hydrolase, as predicted with the extracellular polymeric matrix in Table S1. Some similar GO terms clustered in the two pairwise comparisons and distinctive KEGG pathway enrichments in the two biofilm phases suggested that some of the same genes in the two pairwise comparisons may participate in the inhibitory activity of biofilm growth throughout the biofilm developmental life cycle and that the distinct genes play different roles in the early biofilm formation and mature biofilm in response to AMP-17.

#### 2.5.3. The Network of DEGs Co-Expressed in the Biofilm Forming and Preformed Biofilm Phase

**Figure 8:**
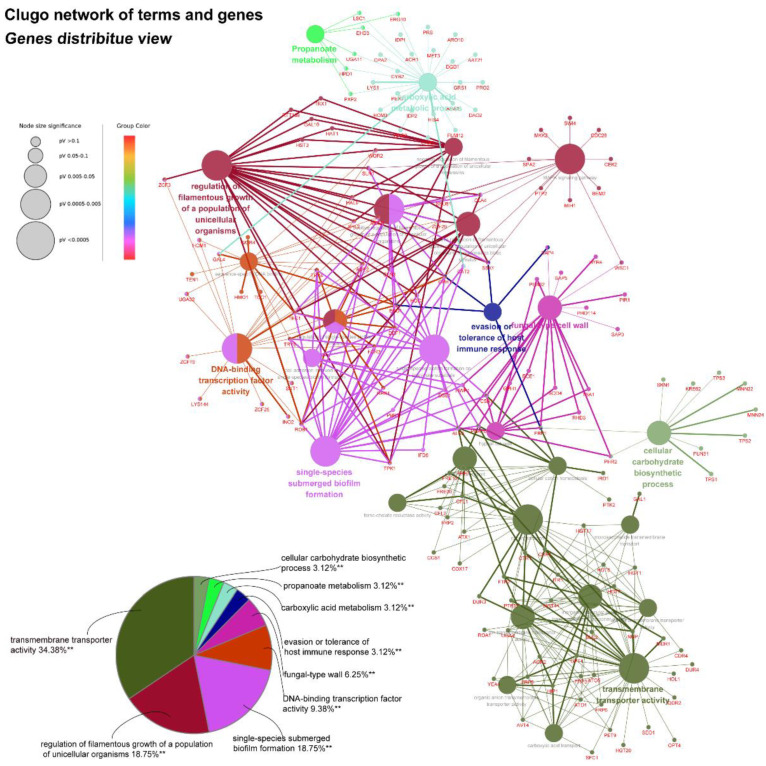
The network of DEG construction and analysis. The Clugo network of terms for DEGs co-expressed in the two pairwise comparisons. Overlapping targets were enriched based on the GO enrichment and the KEGG signaling pathways which clustered into the nine groups. Kappa score = 0.4 was considered. ** *p* < 0.01 were obtained for control versus treated samples.

Previous work has already analyzed 325 DEGs upregulated or downregulated in both the biofilm-forming and mature biofilm states after AMP-17 treatment (Figure S2). Therefore, we hypothesized that these DEGs might play a critical and continuous role in the inhibitory effect of AMP-17 on the whole biofilm developmental life cycle. Consequently, we filtered the unknown functional genes and selected 252 DEGs for further analysis. The Clugo session of Cytoscape was applied to enrich the main term or pathway and search for valuable molecules in biofilm inhibition and elimination. As the Clugo network showed, 28 of the most representative terms or pathways were obtained, among which, the first three terms of DEG enrichment were transmembrane transporter activity, regulation of filamentous growth of a population of unicellular organisms, and single-species submerged biofilm formation, with a ratio of 34.38%, 18.75%, and 18.75%, respectively (Figure 8). Furthermore, we found some genes involved in several terms or pathways, which might be the key regulators and probable targets that trigger AMP-17 to inhibit the forming and preformed biofilms. For example, TEC1, presented in several pathways, including the regulation of filamentous growth, MAPK, and DNA binding, has been shown to positively regulate the extracellular matrix composition and biofilm morphology as a transcription factor [28] and has been identified as a significant factor that some antifungal agents target to suppress biofilms in *C. albicans* [29,30]. Therefore, the predicted pathway and target genes analyzed by the network and functional enrichment would facilitate elucidating the probable biological process that AMP-17 regulates in biofilm inhibition and mature biofilm elimination. The gene details of each term or pathway derived from Clugo software are provided in Table S6.

Further, to understand the potential relationship among key DEGs, the interactome of 380 genes (Log_2_FC > 2.0 or Log_2_FC < −2.0) in the biofilm-forming phase depicted the physical interaction within the 190 nodes and 779 edges (interaction score > 0.4) (Figure S3). According to the degree of connectivity based on the statistics of the network, we classified the DEGs into four grades and identified a total of about 15 hub genes in the center of this network. These hub genes are connected to at least 20 other putative targets. Thus, one drug molecularly targeting these hub proteins will likely impact the functioning of a minimum of 20 targets. As the view of the gene-gene network shows, the CDC28 gene (cyclin-dependent protein kinase) might be of interest as it has a higher number of connecting partners (degree = 44). It plays an important role in the MAPK pathway in which Hgc1-cdc28 simultaneously phosphorylates and regulates multiple substrates, controlling multiple cellular apparatuses for the morphogenesis of *C. albicans* [31]. Next, the following higher degree DEGs (LSC1, CYB2, ACH1, GLA4, and ERG10) mainly take part in the carboxylic acid metabolic processes involved in homeostasis and the biosynthesis of amino acids and secondary metabolites, whose metabolism and transport are necessary for filamentous growth and ATP supply [32,33]. Combining the network of gene-gene interaction with the Cluo pathway enrichment of DEGs, it is suggested that AMP-17 suppresses the *C. albicans* biofilm by disturbing the carboxylic acid metabolic activity mediated by the MAPK pathway, and finally causes retardation of filamentous growth and biofilm development. To sum up, these hub genes play a vital role in maintaining the morphogenesis and virulence of the biofilm under adverse conditions for survival and growth, and thus, would be the targets of AMP-17 against *C. albicans* biofilms.

### 2.6. Exposure to AMP-17 Alters the Gene Expression in C. albicans Biofilms

**Figure 9:**
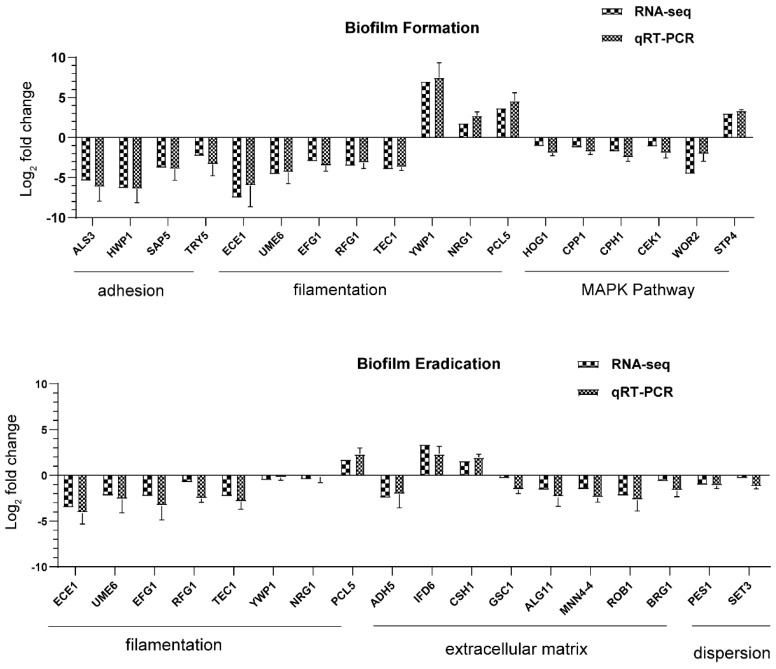
The fold change values of key genes were measured by RNA-seq and qRT-PCR. (**A**) The qRT-PCR gene expression trend of genes involved in biofilm formation was consistent with the RNA-seq analysis under 32 μg/mL AMP-17 treatment. (**B**) The expression trend of genes of preformed biofilm as characterized by qRT-PCR was consistent with that of RNA-seq analysis under treatment with 128 μg/mL AMP-17. All values are based on three independent repeats and are presented as the mean ± SD.

To validate the accuracy of the RNA-seq results, 18 important DEGs in each pairwise comparison were screened for qRT-PCR analysis based on their function or annotation (Figure 9). First, as the Log_2_FC level of selected genes in the biofilm-forming phase shown in Figure 9A, four adhesion-related genes, ALS3, HWP1, SAP5, and TRY5, were downregulated after 32 μg/mL AMP-17 treatment by 6.17-, 6.40-, 3.93-, and 3.35-fold, respectively. The downregulation of adherence genes indicated that AMP-17 suppressed initial biofilm formation, mirroring the inhibitory effect of the peptide on the adhesion of *C. albicans* (Figure 4). Significant hyphal initiation regulators are required for the long-term maintenance of filamentous growth and cell elongation; these regulator genes, ECE1, UME6, EFG1, and TEC1, were dramatically downregulated by 4.33-, 3.50-, 3.17-, and 3.69-fold, respectively. On the other hand, NRG1, encoding a negative regulator of hyphal growth, was found to be upregulated by 2.75-fold in response to AMP-17. Yeast wall protein YWP1- and Putative cyclin PCL5-associated filamentous growth were also 7.48- and 4.58-fold upregulated, respectively. The downregulation of hyphal growth and the upregulation of the hyphal growth repressor may collectively be responsible for the inhibitory effect of AMP-17 on filamentation. In addition, the KEGG enrichment results of DEGs showed that the most obvious enrichment pathway was the MAPK pathway in the biofilm-forming phase after AMP-17 treatment. In *C. albicans*, the HOG-MAPK pathway regulates important physiological functions such as morphology, oxidative stress pressure, cell wall biosynthesis, and yeast-to-hyphae transition [34]. Some genes involved in the MAPK pathway (HOG1, CKE1, CCP1, CPH1, and WOR2), with AMP-17 treatment, were downregulated by 1.94-, 1.94-, 1.78-, 2.48-, and 2.06-fold, respectively, and STP4 was upregulated by 3.35-fold. These differently expressed genes suggest AMP-17 might inhibit adhesion and filamentous growth through the MAPK pathway, contributing to biofilm repression.

In regard to the inhibitory effect of AMP-17 on mature biofilm at the gene level, some genes of 128 μg/mL AMP-17-treated preformed biofilm presented the same regulation as the forming biofilm (Figure 9B, Table S1). Meanwhile, certain genes with specific functions were also chosen to carry out qRT-PCR experiments for validating the sequencing results. In terms of filamentous regulation, ECE1, UME6, EFG1, and TEC1 were also downregulated and PCL5 upregulated after AMP-17 treatment, but with a minor downregulation or upregulation compared to the AMP-17 treatment of forming biofilm. However, two important gene-encoding negative regulators of hyphal growth (NRG1 and YWP1), were not found to be statistically regulated in response to AMP-17, indicating AMP-17 played a weaker role in hyphal growth, ascribed to the fact that filamentous growth nearly stops once biofilms mature. Additionally, a mature *C. albicans* biofilm is encased in an extracellular matrix [35], a contributing factor to the high degree of resistance to antimicrobial drugs. Hence, a good inhibitory effect on mature biofilm likely depends on the destructive ability of the biofilm matrix. As Figure 9B showed, seven relevant genes regulating extracellular matrix, ADH5, ALG11, MNN4, ROB1, BRG1, and GSC1, were downregulated by 2.08-, 2.40-, 2.45-, 2.72-, 1.68-, and 1.60-fold, respectively. While IFD6, which encodes alcohol dehydrogenases and is a negative regulator of matrix production, was upregulated by 2.73-fold. In addition, CSH1, another negative regulator, was upregulated by 1.99-fold. Moreover, yeast cell dispersion in *C. albicans* biofilm was considered an important factor causing invasive infection. In this study, two genes related to dispersion, PES1 and SET3, were downregulated in the group of AMP-17-treated preformed biofilm. Therefore, the expression level of these specific genes suggests the antibiofilm effect of AMP-17 on mature biofilms was likely closely associated with filamentation repression, extracellular matrix damage, and cell dispersion inhibition, in the view of transcriptional regulation. In general, the qRT-PCR results were consistent with those from RNA-seq and confirmed the reliability of RNA-seq.

## 3. Discussion

*C. albicans* is one of the leading causes of hospital-acquired bloodstream infections. When patients undergo immunosuppressive therapies, organ transplantation, and chemotherapy, they are susceptible to systemic *C. albicans* bloodstream infections, also known as candidemia and tissue colonization [36]. Nevertheless, the presence of *C. albicans* biofilms makes the current problems more serious because the biofilm form is less sensitive to common antifungal drugs, such as azoles and conventional amphotericin B, than planktonic cells [37,38]. Furthermore, *C. albicans* biofilms are dynamic and highly structured three-dimensional networks composed of a large number of hyphae and an extracellular matrix, which worsen the problem. Given the severity of biofilm-related diseases, it is urgent to find alternative antifungal agents that can efficiently control biofilms. Interestingly, some antimicrobial peptides (AMPs) have been found to have a broad-spectrum antimicrobial ability and excellent antibiofilm capacity, according to previous reports [39,40]. In our previous study, AMP-17 showed a strong in vitro inhibitory effect toward multiple fungi of reference strains and clinical isolates, including *C. albicans*, *C. neoformans*, *C. glabrata*, and *C. tropicals*, while it was not toxic towards human erythrocyte cells [17,18,19]. In the current study, AMP-17 showed strong antibiofilm activity against early-forming biofilm and mature biofilm. In addition, the CV assays also displayed a similar inhibitory effect of the peptide against *C. albicans* in both the biofilm-forming and mature biofilm phases (Figure 1). Interestingly, the antibiofilm activity of AMP-17 was stronger than that of FLC at the same concentration. This effect was further confirmed through morphological studies using the SEM and CLSM assays (Figure 2 and Figure 3). Morphological analysis validated the specific 3D network was hardly formed and the structure was damaged in response to AMP-17. As biofilm infections are too stubborn to control effectively in clinical practice, we were inspired to elucidate the potential molecular mechanisms of AMP-17 and to provide an alternative candidate agent for candidiasis therapy.

The formation of *C. albicans* biofilms involves several specific stages, including the early, developmental, and mature phases. Cell adherence is the first step in biofilm formation, followed by the *C. albicans* yeast cell gradually transforming into hyphal forms, which is essential not only for biofilm growth but also for biofilm dissemination. Repression of adhesion or the yeast-to-hyphae transition can cause biofilm formation defects, making a novel target for biofilm-specific treatment. Our data demonstrated that AMP-17 not only suppressed adhesion effectively but also inhibited the yeast-to-hyphae transition. In terms of inhibitory adhesion, our data showed that 32 μg/mL AMP-17 remarkably removed more than 50% of adhering cells or adhered cells from the microplate surface (Figure 4). To the best of our knowledge, relative hydrophobicity is considered a pathogenic factor contributing to *Candida* spp. adherence on the host surface [24]. We also observed that 64 μg/mL AMP-17 significantly reduced the hydrophobicity index. Therefore, the inhibition of adhesion in AMP-17 treatment was likely due to the decrease in hydrophobicity induced by the peptide, contributing to the earliest antibiofilm effect in the *C. albicans* biofilm life cycle. While, in terms of the inhibitory yeast-to-hyphae transition, the filamentation assay showed that AMP-17 obviously suppressed the morphological transition (Figure 5). Starting at 8 μg/mL of AMP-17, only smooth-edged colonies were found on solid Spider medium. At 32 μg/mL, filamentous cells were hardly seen in the RPMI 1640—10% FBS medium, and yeast-like cells were locked in the hyphae-induced medium. Nevertheless, the inhibition of adhesion and yeast-to-hyphae in the AMP-17 treatment was stronger than that produced by FLC treatment at the same concentration. The antibiofilm action of AMP-17 seemed to be the inhibitory adhesion or yeast-to-hyphae transition.

Adhesion is thought to be the first event in hyphal growth, and a family of cell surface proteins known as agglutinin-like sequence (Als) proteins are associated with the cell adhesion and aggregation of yeast cells and are an essential component of biofilm formation [41]. RNA-seq results showed that the relevant Als family genes including ALS1, ALS2, ALS3, ALS4, and ALS7 were downregulated, as expected (Table S4). Among these genes, ALS3 reduction was greater than others, with a downregulation of 5.35-fold. Furthermore, the downregulation of other adhesion-related typical genes such as HWP1, SAP5, and TRY5 [42,43] was also evidenced to reduce the cell adhesion and virulence ability, contributing to the detachment of biofilms from an abiotic substrate (Table S1, Figure 9). On the other hand, hyphal elements contribute substantially to the *C. albicans* biofilm volume, and the lack of this morphological form in AMP-17 treated biofilms was evidenced by a sparse, preliminary biofilm with scattered blastopores shown in SEM and CLSM assays (Figure 2 and Figure 3). In addition, the expression of hyphal initiation (EFG1, CPH1, and TEC1) and long-term maintenance (UME6, HGC1, and ECE1) stages were downregulated after AMP-17 treatment. Reportedly, Efg1 and Cph1, the first identified regulators of hyphal development, synergistically regulate virulence genes, while Tec1 has been shown to regulate hyphal development and virulence in *C. albicans* [42]. Furthermore, genes negatively regulating biofilm formation were upregulated when exposed to AMP-17 in the current study, such as the yeast form-specific gene (YWP1) and genes encoding a repressor of the filamentation program (NRG1) [44,45]. As yeast-to-hyphae and filamentous development are necessary for the structure of 3D biofilms, these filamentation-regulated genes in the mature biofilm phase are also altered similarly to the biofilm-forming phase when treated with AMP-17. Consistently, 18.75% of genes participate in the regulation of filamentous growth in the co-expressed DEGs of both AMP-17-treated forming biofilms and mature biofilms (Figure 8A). This phenomenon suggests that the repression of AMP-17 on hyphae took place during the whole biofilm developmental period. Above all, the downregulation of adhesion-related or filamentous-associated genes or upregulation of the negative regulators contributed to defective *C. albicans* biofilm or its inability to form a biofilm, which powerfully explains the inhibitory effect of AMP-17 on biofilms. Importantly, the hyphae-related antibiofilm activity of AMP-17 in C. albicans is likely to produce a profound effect on the drug-resistant *Candida* spp. Due to drug resistance, echinocandin occasionally exhibits reduced antibiofilm activity in *Candida* spp. with an *fks* mutation [6], while AMP-17 might overcome the difficulties in treating echinocandin-resistant *Candida* spp., leading to an increased interest in a future investigation into the synergist effect of the peptide against *C. albicans* biofilm in combination with an echinocandin.

From the regulatory-pathway perspective, since the key transcriptional factor regulating hyphal growth (EFG1) involved in the RAS/cyclic AMP (cAMP) pathway was downregulated after AMP-17 treatment, as the RNA-seq and qRT-PCR results showed, we further verified the expression of the main genes in this pathway by RNA-seq, including RAS1, CYR1, PDE2, and EFG1. The results showed that, except for EFG1, all these genes remained unchanged. Therefore, AMP-17 treatment exerted a minor influence on the Ras/cAMP pathway. Nevertheless, KEGG enrichment showed that a large number of genes in the MAPK pathway were altered greatly in response to AMP-17 (Figure S4). Previous reports suggested that MAPK participates in cell wall forming under vegetative and filamentous growth, playing an important role in morphogenesis [25,46]. The gene expression level of HOG1, CEK1, CCP1, CPH1, and WOR3 was found to be downregulated after AMP-17 treatment. As one of the MAPKs, Cek1 has a role in the biofilm formation and filamentous growth of *C. albicans*. While another MAPK, Mkc1, has a function in the formation and maintenance of cell wall structure [25]. Our data showed that CEK1 was downregulated and MKC1 nearly unchanged, indicative of hyphae inhibition mediated by the Cek1-MAPK pathway other than Mkc1 under AMP-17 treatment. Furthermore, some antifungal agents exert antibiofilm effects by targeting cell walls or membrane components, such as FLC, which exerts its antifungal activity by interfering with the ergosterol biosynthesis pathway [47]. Hence, the inhibition of ergosterol biosynthesis by AMP-17 may also account for its antibiofilm effect. As we found, five ergosterol biosynthetic genes (ERG1, ERG27, ERG251, ERG4, and ERG6) were collectively upregulated after AMP-17 treatment, which directly resulted in the reduction in ergosterol biosynthesis (Figure S4). Blocking ergosterol biosynthesis could be effective in both yeasts and biofilms, and the depletion of ergosterol directly changes cell membrane functions, which eventually leads to cell death. This explained the increased numbers of dead hyphae and cells under AMP-17 treatment, illustrated by the CLSM images (Figure 2). Collectively, we postulate that the antibiofilm effect of AMP-17 is due to its regulation of the MAPK pathway and suppression of ergosterol biosynthesis metabolism, which in turn led to abnormal morphogenesis and cell death, and finally, inhibited biofilm development.

As is well known, a salient feature of the *C. albicans* biofilm is the presence of the biofilm matrix composed of major macromolecules including polysaccharides, proteins, and nucleic acids, which plays a preponderant role in protecting the biofilm cells from antifungal drug treatment [8]. The AMP-17 treatment inhibited the expression of several genes required for encoding enzymes that produce polysaccharide components (e.g., ALG11 and MNN4) in the mature biofilm phase, indicative of the repression of biofilm matrix production [48]. In the AMP-17-treated preformed biofilm group, there was a reduction in GSC1 involved in the β-1,3-glucan synthesis, resulting in broad defects in the matrix synthesis. Interestingly, a few genes encoding alcohol dehydrogenases, such as Adh5, Csh1, and Ifd6, play roles in matrix production: Adh5 acts positively, while Csh1 and Ifd6 act negatively [49]. In the current study, the AMP-17-treated preformed biofilms exhibited a higher gene expression of CSH1 and IFD6, and lower expression of ADH5, compared to the untreated samples. ADH5 encodes alcohol dehydrogenases that may promote the entry of ethanol into the TCA cycle for energy or via the glyoxylate shunt to provide hexose for β-1,3 glucan synthesis, while CSH1 and IFD6 act preferentially to yield a matrix inhibitory signal, although the roles of such genes may be too complex to reveal how AMP-17 regulates the *C. albicans* biofilm matrix production or degradation. Indeed, the changes in these matrix-regulated genes gave us a glimpse that the inhibition of matrix production with AMP-17 treatment plays a critical role in antibiofilm activity, particularly in mature biofilm, due to their impact on carbon metabolism. Sarah et al. studied that the TCA cycle plays a vital part in mediating biofilm formation, especially by influencing the matrix composition in *S. aureus* [50]. As a result, the pathways of the TCA cycle, phenylamine metabolism, propanoate metabolism, and beta-Alanine metabolism, enriched in the AMP-17 treated preformed biofilms (Figure 7) evidenced the interference of the peptide with matrix production and biofilm development. On the other hand, the dispersal of *C. albicans* into the surrounding environment (primarily as round, yeast-form cells) occurs throughout biofilm formation, with great numbers of cells being dispersed once the biofilm reaches maturity. Therefore, it is necessary for antifungal agents to prevent the *C. albicans* cells within biofilms from dispersing or disseminating. Set3, a component of a chromatin-modifying complex, is also required for dispersal, probably by being recruited to specific genes by the transcriptional regulator Nrg1. In response to AMP-17, some genes linked to dispersals, such as NRG1, PES1, and SET3, were suppressed in the mature biofilm phase (Figure 9). Overexpression of either NRG1 or PES1 increased the number of cells released from the biofilm. Therefore, the inhibition of filamentous growth, biofilm matrix production, and cell dispersal may account for the elimination activity of AMP-17 on preformed biofilm.

In addition, AMP-17’s antibiofilm activity might also be achieved through a multitarget action, as considerable DEGs are involved in carbohydrate metabolism, fatty acid metabolism, nucleotide metabolisms, and amino acid metabolism. For example, genes encoding carboxylic acid metabolic proteins (CDC28, LSC1, CYB2, ACH1, and GLA4) [51] and metal ion transport (CTR1, CFL1, CFL2, and CRP1) [52], required for various metabolic or catalytic activities, were altered greatly after AMP-17 treatment. Furthermore, as the necessary components of the cell membrane and cell skeleton, the abnormal metabolism of carbohydrates, glycerophospholipid, and various amino acids certainly affects the organism’s life cycle, including cell integrity and biofilm growth. Previous studies have revealed a link between the abnormal cell cycle and cell death of AMP-17-treated planktonic cells [20]. Hence, the significant antibiofilm effect of AMP-17 not only depends on the repression of adhesion, yeast-to-hyphal, matrix production, and dispersion but also on the damage to yeast cells in the biofilm.

## 4. Materials and Methods

### 4.1. Antimicrobial Peptide

AMP-17 was obtained from the recombinant expression plasmid pET-28a (+)—(AMP-17) using a prokaryotic expression system and purified with Ni-NTA beads (Novagen, Germany) [17,20]. Briefly, *E. coli* BL21 (DE3) harboring the plasmid was incubated at 37 °C in liquid luria broth (LB) medium containing 100 μg/mL kanamycin with shaking at 200 rpm and until its OD_600_ reached 0.5. Then, a final concentration of 0.05 mmol/L IPTG was added, and the culture was incubated for 24 h at 32 °C. Finally, bacterial cells were collected by centrifugation (12,000 rpm for 10 min) at 4 °C, resuspended in lysis buffer (50 mM Tris-HCl [pH 8.0], 1 mM EDTA, and 100 mM NaCl), and disrupted by sonication (160 W, ultrasonic for 1 s, pause for 2 s, 3 min). Subsequently, the lysate was dissolved in urea, the prepared protein was purified through His Tag’s Ni-NTA affinity chromatography, and the imidazole was removed by ultrafiltration using an ultrafiltration tube. AMP-17 was granted a Chinese patent license in 2019 (patent number: ZL 2016 1 0428119. 8) and the amino acid sequence was shown in our previous report [20].

### 4.2. Strains and Growth Condition

A *C. albicans* reference strain SC5314 was preserved by the Key Laboratory of Modern Pathogenic Biology of Guizhou Medical University. *C. albicans* were grown at 35 °C in YPD medium (1% yeast extract, 2% peptone, and 2% dextrose) and stored on YPD plates solidified with 1.5% agar at 4 °C. A single *C. albicans* colony was inoculated into YPD broth and incubated at 35 °C for 12 h with shaking at 200 rpm and grown to the exponential phase for further experiments. To stimulate the hyphal growth of *C. albicans*, RPMI-1640 (Invitrogen, Carlslad, CA, USA) supplemented with 10% fetal bovine serum (Sigma-Aldrich, St. Louis, MO, USA) was used as the culture medium.

### 4.3. Minimal Inhibitory Concentration (MIC) of Planktonic C. albicans

The minimum inhibitory concentration (MIC) for AMP-17 against *C. albicans* was tested by a broth micro-dilution assay in 96-well microtiter, according to the procedures of the Clinical and Laboratory Standards Institute (CLSI) standard, with slight modifications [53]. Briefly, the exponential fungal cells incubated in YPD medium overnight were diluted to 1–2 × 10^3^ CFU/mL and co-incubated with various concentrations of AMP-17 in a Sabouraud-dextrose broth (SDB) at 35 °C for 24 h. The lowest concentration at which no visual growth was observed by the naked eye was considered the MIC value. Specifically, FLC was used as a positive control drug and *Candida*. *parapsilosis* ATCC22019 as the quality control strain. When the MIC of FLC against *C. parapsilosis* ATCC22019 was between 0.5 μg/mL and 4.0 μg/mL, the experimental data were reliable. The experiments were repeated in triplicate and three multiple wells were set up in each experiment.

### 4.4. Biofilm Formation

As fetal bovine serum (FBS) is needed for filamentous growth during *C. albicans* biofilm formation, FBS was added to RPMI-1640 (RPMI 1640-FBS) at a final concentration of 10% to guarantee biofilm development. Briefly, yeast cells at a density of 1 × 10^6^ CFU/mL were seeded into a flat-bottomed 96-well microplate of polypropylene at 37 °C for 90 min. Then, non-adhered cells were removed by washing the walls twice using sterile PBS, followed by RPMI 1640-FBS. Next, the adherent yeast was co-incubated with AMP-17 for 12 h at 37 °C to detect the effect of the peptide on biofilm formation. In order to observe the eradication ability of AMP-17 on mature biofilm, the culture needed to be incubated for 24 h at 37 °C to grow mature, and then was co-incubated with AMP-17.

### 4.5. XTT Reduction Assays

The XTT/Menadione assay was applied to assess the biofilm mass of *C. albicans* and determine the antibiofilm efficacy of AMP-17 on biofilm formation and mature biofilm, measured by the microtiter plate reader at 490 nm [29]. Before each assay, the XTT salt [2,3-bis (2-methoxy-4-nitro-5-sulfophenyl) 2H-tetrazolium-5-carboxanilide sodium salt] was dissolved in Ringer’s solution at a final concentration of 0.5 mg/mL. Notably, a menadione solution at a final concentration of 0.4 nM was prepared and filter-sterilized. Finally, the XTT solution was mixed with the menadione solution at a ratio of 20:1 (*v*/*v*). At the end of the incubation period, the biofilm was washed with sterile PBS to remove planktonic cells, followed by 100 μL of freshly prepared XTT solution added to each wall and incubated for 30 min in the dark at 37 °C. The supernatants were transferred to a new flat bottom plate and an OD490 nm, which represents the metabolic activity of the *C. albicans* biofilm, was detected at 490 nm.

### 4.6. Crystal Violet (CV) Assays

In addition to the XTT reduction method, CV assays that represent biofilm biomass are also usually used to evaluate biofilm viability. Once the samples were finished, they were washed twice using sterile PBS to remove non-adhered cells, and then stained with 5% crystal violet (Solarbio, Beijing, China) for 10 min. Next, the stained biofilms were washed gently with sterile PBS, and destained with 95% ethanol; then, 100 μL PBS was added to every microplate wall. Finally, the absorbance of each well was measured at a wavelength of 560 nm.

### 4.7. Detection of Biofilm Inhibition and Biofilm Eradication

*C. albicans* biofilm formation in the presence of AMP-17 was studied in accordance with the guidelines provided by the Clinical and Laboratory Standards Institute (CLSI) for antifungal susceptibility testing. For the biofilm inhibition of AMP-17, *C. albicans* suspension on logarithmic phase was diluted to 2 × 10^6^ CFU/mL in RPMI medium and incubated with 2-fold serial dilutions of AMP-17 in a 96-wall flat-bottomed microplate at 37 °C for 12 h. After incubation, the supernatant was removed without disturbing the intact biofilms and washed twice with sterile PBS buffer to remove any planktonic cells. Finally, the biofilm biomass was measured by XTT reduction assays, and the biofilm viability was analyzed with the following equation:

$$\mathrm{Biofilm}\;\mathrm{formation}\left(\%\right)=\left(\frac{A490_{490}^{\mathrm{treated}}-A490_{490}^{\mathrm{medium}}}{A490_{490}^{\mathrm{untreated}}-A490_{490}^{\mathrm{medium}}}\right)\times 100$$

For biofilm eradication, mature biofilms needed to be prepared as previously described. Then, the preformed biofilm was washed without disturbing the formed biofilm and incubated with various concentrations of AMP-17 for 12 h at 37 °C. The subsequent processing of AMP-17-treated preformed biofilm was the same as the above biofilm formation.

### 4.8. Cell Surface Hydrophobicity Test

A cell surface hydrophobicity (CSH) assay was performed to assess the cell adhesion of *C. albicans*. The logarithmic phase of *C. albicans* was diluted to 1.0 × 10^7^ CFU/mL in YPD medium and treated with AMP-17 at 35 °C for 24 h. The cells were harvested, resuspended using sterile PBS, and the optical density (OD) values were measured at 600 nm (*A*_0_). Chloroform was added to the suspension, vortexed for 1 min, and settled at room temperature for 30 min. The upper aqueous phase was collected and the OD_600_ (*A*_1_) was determined. The CHS of *C. albicans* cells was quantified by the hydrophobicity index using the following equation:

(1) $$\mathrm{hydrophobicity}\;\mathrm{index}=1-\frac{A_{1}}{A_{0}}\times 100\%$$

### 4.9. Cell Adhesion Assay

Efficient adhesion is the first step to attaching closely to the host surface and plays an important role in biofilm formation. In vitro cell adherence was performed as previously reported, with some modifications [29,54]. Briefly, *C. albians* cells cultured on the exponential phase were diluted to 2 × 10^6^ CFU/mL and co-incubated with AMP-17 in an RPMI medium in a 6-well flat-bottomed microplate at 37 °C for 90 min. Subsequently, the walls were washed using sterile PBS to remove the non-adherent cells and scraped using a cell scraper. Next, the suspension containing adhered cells for each group was serially diluted, and the diluted suspension (5 μL) was spot-seeded on a YPD plate, and the colonies were counted after 24 h incubation. For the inhibitory effect of AMP-17 on preformed adhesion, a *C. albicans* suspension was previously cultured for 90 min until the cells adhered to the surface of the microplate, co-incubated with AMP-17 for another 90 min, and finally washed twice. Finally, the number of adhered cells was counted as per the previous description.

### 4.10. Yeast-to-Hypha Transition Assay

The effect of AMP-17 on the transition of yeast-to-hyphal in *C. albicans* was performed according to the previous report with minor modifications [29,42]. *C. albicans* cells were diluted to 2 × 10^5^ CFU/mL and co-incubated with AMP-17 in an RPMI-FBS medium in a 12-well flat-bottomed microplate at 37 °C for 2, 4, and 6 h. Images of cells were captured using an inverted microscope (IX51, Olympus, Tokyo, Japan). To test the inhibitory effect of AMP-17 on hyphal formation, *C. albicans* cells were treated with AMP-17 for 2 h and it was observed whether the yeast cells sprouted. At least 200 *C. albicans* cells were counted to quantify the hyphal formation rate, and Image J was applied to measure the length of the hyphae. In addition, the *C. albicans* filamentation assay was carried out on a solid Spider agar plate. A total of 10 μL of *C. albicans* suspension (1 × 10^6^ CFU/mL) was added to the plate containing AMP-17 or FLC and incubated at 37 °C for 3 days. Images of the colony edges were obtained using a microscope.

### 4.11. Confocal Laser Scanning Microscope (CLSM) Assays

A confocal laser scanning microscope (CLSM) was applied to visualize the *C. albicans* biofilm. Briefly, sterile polylysine-treated cover slips with an area of 1 cm^2^ were used as the carrier and placed in a 12-well plate. Following the method described above, the *C. albicans* biofilm was incubated for 30 min at 37 °C in the dark with 10mM PI (Sigma, St. Louis, MO, USA) and 10 mM SYTO9 (Invitrogen, USA). Next, the slips were washed, fixed with paraformaldehyde, and stuck on a sterile polylysine-treated glass slide. Finally, images were taken with an Olympus CLSM (Markham, ON, Canada) at an excitation wavelength of 488 nm for SYTO9 and 525 nm for PI. SYTO9 could move through the membrane of viable cells, resulting in the accumulation of green fluorescence, while PI stained the dead cells, emitting red fluorescence.

### 4.12. Scanning Electron Microscopy (SEM)

Scanning electron microscopy (SEM) was applied to evaluate the morphological surface changes of the biofilms. A sterile polylysine-treated cover glass as a biofilm carrier was placed in a 6-well plate and *C. albicans* biofilms were cultured according to the above method. After incubation, the samples were washed twice in PBS and then fixed with 2.5% glutaraldehyde at 4 °C overnight. Next, the fixed samples were washed with PBS, followed by dehydration using a tertiary butyl alcohol series (50, 75, 95, and 100%) for 10 min. Subsequently, the samples were dried in a high vacuum evaporator and coated with a thin layer of gold-palladium. Finally, the prepared sample was placed on the Hitachi H-7650 Scanning Electron Microscope (Tokyo, Japan) for observation and to obtain images.

### 4.13. Quantitative Reverse Transcription Polymerase Chain Reaction (qRT-PCR)

qRT-PCR assays were performed to verify the accuracy of RNA-seq. Biofilm samples were collected in a 1.5 mL EP tub (Axygen, Sigma-Aldrich, St. Louis, MO, USA) of free-RNase with a cell spatula. Total RNA was extracted using Trizol and quantified using a NanoDrop Spectrophotometer (ND-200, Thermo Scientific, Waltham, MA, USA). Then, the RNA was reversed to cDNA in a 20 μL reaction mixture using a PrimeScript RT reagent Kit with gDNA Remover (Takara, Maebashi, Japan), with procedures as follows: gDNA removal reaction at 42 °C for 2 min, followed by a reverse transcriptional reaction at 42 °C for 2 min, and 85 °C for 5 sec to generate cDNA. Primers of *C. albicans* for qRT-PCR were designed using Primer Premier 5.0 and synthesized by Sangon Biotech. According to the SYBR Premix Ex Taq TM Kit (Takara, Japan) protocol, the qRT-PCR mixture (20 μL) was freshly prepared, containing SYBR green fluorescent dyes, PCR reverse primer, cDNA, and RNase-free water. The qRT-PCR process was performed using an ABI7300 fluorescence quantitative PCR system with the following cycles: 95 °C for 30 s for pre-denaturation, along with 95 °C for 5 s and 60 °C for 30 s for a total of 40 cycles. A 20 μL reaction system was used in the qRCR system. All data were normalized to the internal reference gene ACT1. The relative quantification of gene expression was computed using the 2^−ΔΔ*Ct*^ method, in which ΔCt = Ct target gene-Ct internal reference genes. The primer sequences are provided in Table S2.

### 4.14. RNA-Seq

#### 4.14.1. RNA Isolation, Library Construction, and Sequencing

RNA-Seq was performed using an Illumine HisSeq System by BGI Tech Company (Shenzhen, China). For the inhibitory effect of AMP-17 on biofilm formation in RNA-seq analysis, the exponential *C. albicans* (2 × 10^6^ CFU/mL) was co-incubated with 32 μg/mL AMP-17 using RPMI medium in a 6-well flat-bottomed plate at 37 °C for 12 h, harvested in a 1.5 mL EP tub, and stored at −70 °C after liquid nitrogen freezing. In order to test the eradication effect of AMP-17 on preformed biofilm, the exponential *C. albicans* (2 × 10^6^ CFU/mL) was incubated in RPMI medium in a 6-well flat-bottomed plate at 37 °C for 24 h to grow mature before being exposed to 128 μg/mL AMP-17 for 12 h. Subsequently, biofilm samples were collected as in the previous step. Meanwhile, untreated biofilm or untreated preformed biofilm were set as controls and each group was prepared with three parallel replicates. Later, all the samples were sent to BGI Corporation for further RNA-seq detection and analysis via the BGISEQ-500 sequencer. The total RNA extraction, quantification, qualification, cDNA library construction, and transcriptome sequencing were performed by Beijing Genomics Institute (BGI) Co., Ltd. (Shenzhen, China). Finally, the platform converted the sequenced image signals into textual signals and stored them as raw data in the FASTQ format.

#### 4.14.2. RNA-Seq Data Analysis

The raw reads generated from sequencing were cleaned by removing adaptor-polluted reads, reads with unknown sequence “N” accounting for more than 50%, and low-quality reads (more than 20% of bases with a Phred threshold score < 15). The clean reads were mapped to the *C. albicans* SC5314 genome (https://www.ncbi.nlm.nih.gov/gene/?term=candida+albicans+sc5314 accessed on 12 March 2021) using the Hierarchical indexing for spiced alignment of transcripts (HISAT) v2.1.0 software [55]. Reads per kilobase million mapped reads (RPKM) were used to access gene expression levels to eliminate the bias caused by the effects of sequencing depth and gene length. Differentially expressed genes (DEGs) were identified with a threshold of Q-value (adjust *p*-value) ≤ 0.001 and log2(fold change) ≥ 1. Gene ontology (GO) and Kyoto Encyclopedia of Genes and Genomes (KEGG) functional classifications were carried out using Blast2GO and KOBAS for annotation of the functions of DEGs (http://kobas.cbi.pku.edu.cn accessed on 18 May 2022). The *p*-value was corrected by the false discovery rate (FDR). The pathway with FDR < 0.01 was considered to be significantly enriched. Data mining and figure presentation processes were analyzed by BGI’s in-house customized data mining system called Dr. Tom (http://biosys.bgi.com accessed on 22 April 2021). The gene interaction network including selected DEGs and neighbors was created by STRING (version 10) and visualized by Cytoscape (version 3.6.1). Default parameters (score value above two and at least four nodes) were used as the cutoff criteria for network module screening. The enrichment analysis of clusters was qualified with a threshold of *p* < 0.05, FDR-adjusted. Finally, DEGs owing to specific functions were selected for qRT-PCR analysis to validate the data from RNA-Seq.

### 4.15. Statistical Analysis

Figures depicting the differential gene expression between AMP-17 treated and untreated groups were created using the DESeq2 package in RStudio. Gene interaction networks were created using the ClueGo application in Cytoscape (http://cytoscape.org, accessed on 13 December 2021).

All other graphs and analyses were carried out in GraphPad Prism (version 7, La Jolla, CA, USA). Every experiment was independently performed at least three times, and the data were expressed as the mean ± standard deviation (SD) of three independent experiments. Differences between experimental groups were assessed for significance using one-way ANOVA. The * *p* < 0.05, ** *p* < 0.01, and *** *p* < 0.001 levels were considered to indicate statistical significance.

## 5. Conclusions

In summary, the study demonstrated that AMP-17 had excellent antibiofilm activity against biofilm formation and mature biofilms in *C. albicans.* Morphological images of AMP-17-treated samples showed the structure of *C. albicans* biofilms was restrained and damaged. In addition, we found that AMP-17 suppressed adhesion, yeast-to-hypha transition, and filamentous growth. RNA-seq revealed that the majority of DEGs are associated with cellular and metabolic processes, catalytic activity, and DNA binding. Specifically, AMP-17 likely inhibited biofilm formation by interfering with the MAPK, biosynthesis of antibiotics, and steroid pathways. In contrast, the inhibitory effect of the peptide against mature biofilm was likely ascribed to the regulation of AMP-17 in TCA and phenylamine metabolisms and propanoate metabolism pathways. In addition to filamentous suppression, the downregulation of genes related to the extracellular matrix and cell dispersal may have an important role in controlling the mature biofilm of AMP-17 in *C. albicans*. Overall, our work provides a scientific basis for elucidating the peptide’s mode of action on *C. albicans* biofilm. At present, our research group is assembling the target gene-knocked-out strains by the CRISPR technique based on what we screened in this study. Moreover, we are planning to observe the genotypic expression and other related mechanisms in the later stages of this process.

## Data Availability

Sequencing data are available from https://www.ncbi.nlm.nih.gov/sra/PRJNA879946, accessed on 1 September 2023.

## Supplementary Materials

The following supporting information can be downloaded at: https://www.mdpi.com/article/10.3390/antibiotics11111474/s1, Table S1: Important genes in AMP-17 treated C. albicans biofilms compared to their expression in untreated C. albicans cells identified by RNA-seq. Table S2: The primers of genes analyzed by qRT-PCR. Table S3: RNA-seq data summary; Table S4: The detailed information of differently expressed genes of AMP-treated forming biofilms obtained by RNA-seq. Table S5: The detailed information of differently expressed genes of AMP-treated preformed biofilms obtained by RNA-seq. Table S6: The DEGs derived from the gene network using Clugo of Cytoscape. Figure S1: Effects of 8µg/mL of AMP-17 and FLC on colony morphology on solid Spider medium. Figure S2: Venn Diagram map of DEGs between 32 μg/mL AMP-17 treated early forming biofilm and 128 μg/mL AMP-17 treated preformed biofilm. Figure S3: The structure of the gene-gene interaction network based on PPI analysis. Figure S4: The MAPK map of DEGs identified by RNA-seq.

## Abbreviations

AMPs	Antimicrobial peptides
FLC	Fluconazole
CFU	Colony forming unit
XTT	2,3-bis-(2-methoxy-4-nitro-5-sulfophenyl)-2H-tetrazolium-5-carboxanilide salt
MBIC	Minimum Biofilm Inhibitory Concentration
MBEC	Minimum Biofilm Eradication Concentration
CV	Crystal Violet
CLSM	Confocal laser scanning microscopy
SEM	Scanning electron microscopy
HI	Hydrophobicity index
RPMI	Serum-free cell freezing medium
FBS	Fetal bovine serum
qRT-PCR	Quantitative reverse transcription polymerase chain reaction
DEGs	Differentially expressed genes
GO	Gene ontology
KEGG	Kyoto encyclopedia of genes and genomes
MAPK	Mitogen-activated protein kinase
TCA	Citrate cycle
