# Supplementary material for: The Antimicrobial Peptide AMP-17 Derived from Musca domestica Inhibits Biofilm Formation and Eradicates Mature Biofilm in Candida albicans
Source: Antibiotics (Basel). 2022 Oct 25;11(11):1474. doi: 10.3390/antibiotics11111474 (PMC9686669; doi:10.3390/antibiotics11111474)
Supplement: Supplementary file 1 [file antibiotics-11-01474-s001.zip › Figure S1-S4.pdf]

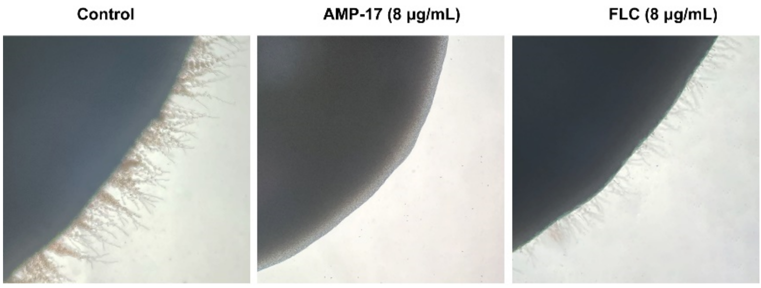

Figure S1. Effects of 8μg/mL of AMP-17 and FLC on colony morphology on solid Spider medium.

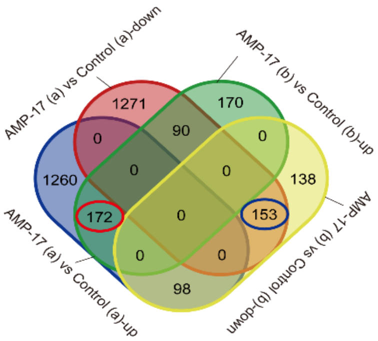

Figure S2. Venn Diagram map of DEGs between 32μg/mL AMP-17 treated early forming biofilm and 128 μg/mL AMP-17 treated preformed biofilm. "a" in the above figure indicated early forming biofilm (12h), while "b" indicated preformed biofilm.

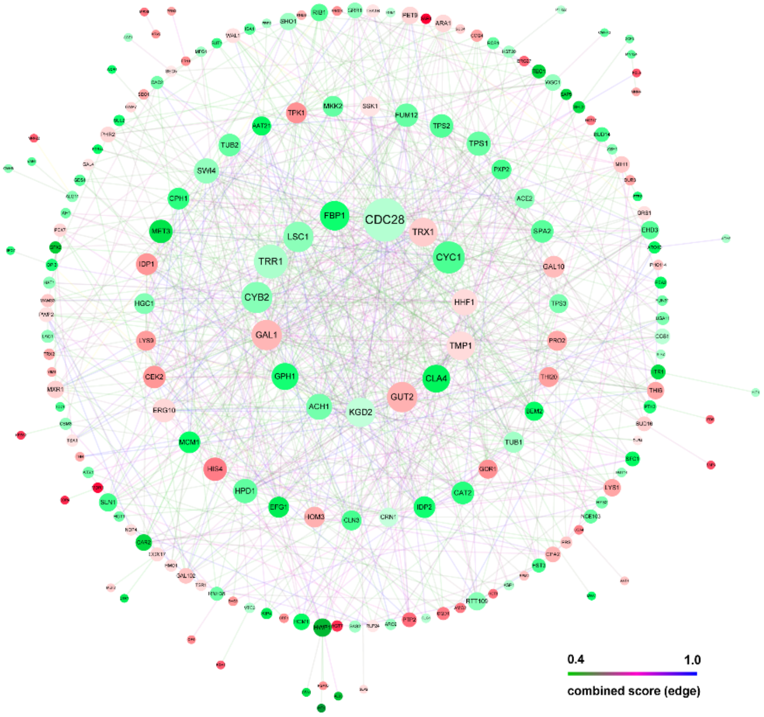

Figure S3. The structure of the gene-gene interaction network based on PPI analysis and degree computation of 380 essential genes. Up-regulated genes were colored in red, and down-regulated genes were colored in green. The 15 hub genes were shown in the center of the network. Hub genes were defined as genes with a degree higher than 20. Nodes represent genes and edges show the interaction with other genes, and the size of the nodes is illustrated in descending order of degree centrality, with a bigger size means the node being more "central".

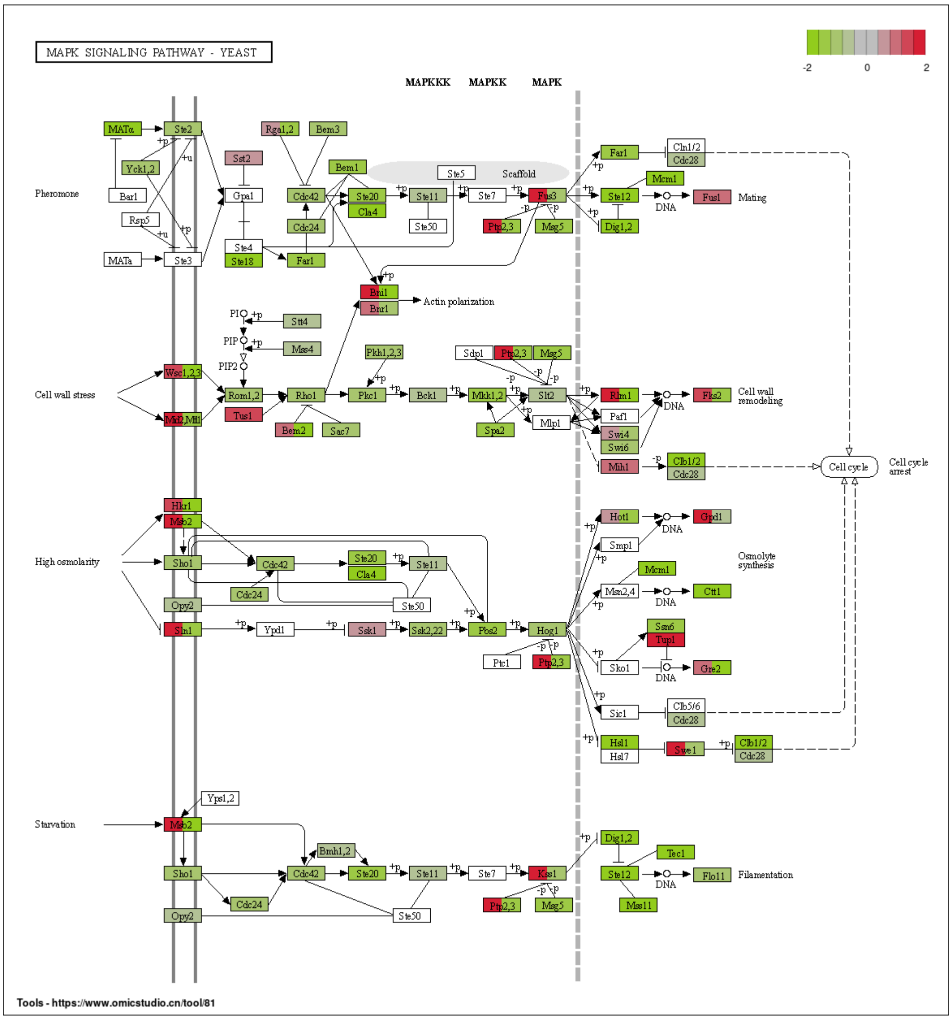

Figure S4. The MAPK map of DEGs identified by RNA-seq in the pairwise comparison of 32μg/mL AMP-17 vs Control in the biofilm forming phrase. The color of DEGs from green to red denoted log2(fold change) of genes ascending gradually from -2 to 2.
