# Supplementary material for: The Antimicrobial Peptide AMP-17 Derived from Musca domestica Inhibits Biofilm Formation and Eradicates Mature Biofilm in Candida albicans
Source: Antibiotics (Basel). 2022 Oct 25;11(11):1474. doi: 10.3390/antibiotics11111474 (PMC9686669; doi:10.3390/antibiotics11111474)
Supplement: Supplementary file 1 [file antibiotics-11-01474-s001.zip › Table S1-S3.pdf]

Table S1. Important genes in AMP-17 treated *C.albicans* biofilms compared to their expression in untreated *C.albicans* cells identified by RNA-seq.

Function and standard or systematic name in CGD[(x)]	Log2FC in expression [(a)]	Log2FC in expression [(b)]	Description[(x)]
**adhesion**			
MCM1	-2.15	-0.81	Transcription factor; regulator of hyphal growth; may act with Wor1p;
TRY5	-2.30	-1.30	regulator of yeast form adherence; required for yeast cell adherence；
ALS3	-5.35	-0.81	Cell wall adhesin; involved in epithelial adhesion and endothelial invasion
HWP1	-6.24	-3.38	Hyphal cell wall protein; host transglutaminase substrate；
SAP5	-3.79	-4.20	Biofilm-specific aspartyl protease; virulence role effected by URA3; biofilm induced;
**Filamentation**			
ECE1	-7.29	-2.94	Candidalysin; cytolytic peptide toxin essential for mucosal infection; hypha-specific
EFG1	-2.93	-2.13	Transcription factor; required for white-phase cell type and hyphal growth
TEC1	-3.97	-2.23	TEA/ATTS transcription factor; white cell pheromone response and hyphal regulation;
UME6	-4.49	-1.23	Zn(II)2Cys6 transcription factor; controls transition to filamentous growth
RFG1	-3.51	-0.76	HMG domain transcriptional repressor of filamentous growth and hyphal genes;
YWP1	6.87	-0.45	Secreted yeast-cell wall protein; involved in adhesion and biofilm formation;
NRG1	1.75	-0.26	Transcription factor/repressor; regulates chlamydospore formation/hyphal gene;
HGC1	-1.02	-1.92	Hypha-specific G1 cyclin-related protein, morphogenesis, biofilm formation;
GAL10	1.13	2.07	UDP-glucose 4-epimerase; galactose utilization; cell wall and filamentation；
STP4	2.91	0.46	C_2H_2 transcription factor; colony morphology-related gene regulation by Ssn6;
PCL5	3.62	1.70	Putative cyclin for Pho85 kinase; Gcn4-induced; rat catheter and Spider biofilm induced
ROB1	-1.26	-2.03	Zn(II)2Cys6 transcription factor; biofilm formation; colony morphology; biofilm induced;
**MAPK Pathway**			
CLA4	-2.53	-0.91	Ste20p family Ser/Thr kinase required for wild-type filamentous growth;
CDC28	-0.62	-1.05	Cyclin-dependent protein kinase; determination of cell morphology; phosphorylation;
PTP2	2.99	2.03	Predicted protein tyrosine phosphatase; regulation of MAP kinase Hog1 activity;
CEK2	1.77	-2.06	MAP kinase required for wild-type efficiency of mating; roles in filamentous growth；
HOG1	-1.06	-0.33	MAPK phosphatase activity involved in regulation of osmosensory signaling pathway;
CEK1	-1.16	-0.69	ERK-family protein kinase; required for wild-type yeast-hypha switch, MAPK pathway;
CPH1	-1.75	1.44	Transcription factor; required for mating, filamentation; pheromone-stimulated biofilms

Function and standard or systematic name in CGD[x]	Log2FC in expression [(a)]	Log2FC in expression [(b)]	Description[(x)]
CCP1	-1.85	-1.02	Cytochrome-c peroxidase N terminus;
WOR2	-4.32	-2.78	Zn(II)2Cys6 transcription factor; regulator of white-opaque switching;
**carboxylic acid metabolic**			
CAR2	-3.83	-2.06	Ornithine aminotransferase; arginine metabolism; biofilm induced;
MET3	-3.32	-3.60	ATP sulfurlyase; sulfate assimilation; biofilm formation;
ARO10	-2.48	-3.42	Aromatic decarboxylase; aromatic alcohol biosynthesis; Spider biofilm induced
CAT2	-1.79	-1.63	Major carnitine acetyl transferase; intracellular acetyl-CoA transport; Hog1-repressed;
PXP2	-1.55	-2.13	Putative acyl-CoA oxidase; enzyme of fatty acid beta-oxidation; biofilm induced；
IFD6	2.94	3.17	Aldo-keto reductase; similar to aryl alcohol dehydrogenases; biofilm induced;
ACH1	-1.15	1.41	Acetyl-coA hydrolase; soluble protein in hyphae; induced on polystyrene adherence;
IDP1	1.78	1.94	Putative isocitrate dehydrogenase; alkaline induced; Spider biofilm repressed;
ADH5	2.35	-0.64	Putative alcohol dehydrogenase; regulated by Nrg1, Tup1; biofilm induced;
**metal ion transport**			
CTR1	-2.88	-3.29	Copper transporter; transcribed in low copper; biofilm induced
CFL1	-2.67	-2.23	Protein similar to ferric reductase Fre10p; negatively regulated by Sfu1p, copper；
CFL2	-3.63	-2.01	Oxidoreductase; iron utilization; biofilm induced;
FRE30	-3.75	-1.95	Protein with similarity to ferric reductases;
FRE7	-3.86	-1.84	Copper-regulated cupric reductase; Spider biofilm induced
CRP1	1.86	3.20	Copper transporter; CPx P1-type ATPase; mediates Cu resistance; biofilm induced
**Ergosterol biosynthesis**			
ERG1	2.27	0.40	Squalene epoxidase, ergosterol biosynthesis; allylamine antifungal drug target;
ERG4	2.62	0.64	sterol C-24 reductase; Mob2p-dependent hyphal regulation; fluconazole-induced;
ERG6	2.40	-0.25	Delta(24)-sterol C-methyltransferase, fecosterol convertation, ergosterol biosynthesis;
ERG27	3.07	1.02	3-Keto sterol reductase of ergosterol biosynthesis; acts in C-4 sterol demethylation;
ERG251	2.54	0.001	C-4 sterol methyl oxidase; role in ergosterol biosynthesis;

[X] As reported in the CGD database (http://www.candidagenome.org/); [(a)] indicated the comparison between AMP-17 treated samples and the control ones in biofilm forming stage. [(b)] indicated the comparison between AMP-17 treated preformed biofilms and the control samples.

Table S2. Primer design for qRT-PCR

Gene name	Foward primer (5' to 3')	Reverse primer (5' to 3')
ACT1	ACCGAAGCTCCAATGAATCC	CCGGTGGTTCTACCAGAAGAG
ALS3	CCATTCGATCCTAACCGCGA	TGGTGCAGTTTTGGTCAGGT
HWP1	GCTGGCTCAAGTGGTGCTAT	TGGCAGATGGTTGCATGAGT
SAP5	AGGGGACGAAATGTTGATGCT	TCATTTGAGCACCTATGGCA
TRY5	CCCTAGTACGTTGCCATCCA	CTTACAGCAGGGTTCGGGTT
ECE1	CCATCATCCACCATGCTCCA	ACAGTAGGTGCTTGGTCAGC
UME6	ACTGCTACTGGATCTGGAGTTGGG	GGTTGGGATTGTGCTTGTTGTTGAG
RFG1	AACAGCAACAGCAGCAACAACAAG	ACCTCCACCTCCACCTCCATTAAG
EFG1	CCGTTGCTGCTGCTACTACTACTG	CACCAGACACATTACTGCCACCAC
TEC1	GCCATGATGTGTACGACCAAGGAG	GTTGTTGTTGTTGTTGTTGTTGCTGTG
YWP1	GCTTCTCCATCTGGTTCCGAATCC	CAGTAGCAGTAGCGGCAGAATCAC
NRG1	ACCAACACCACCACCATATCCAATG	ATGGTATGGCTGAGGTTGTTGTAGTG
PCL5	CAAAGACGAACAACCAACCCTCTTG	AGGCTTTAACCTTCTTGGCAACTGG
HOG1	GGCATACCATGATCCAACCGATGAG	GCCACACCAACAGTTTGATGAAAGTC
CCP1	AAGTCCCTGAAGGTAAAAGTGCTAGTG	CCTGATGTATGCCATGCCAATCTTAAC
CPH1	AAGGACTCCACTACCCCCAA	CACAAACCCGGCATTAGCAG
CEK1	CTCAAGCACAACAACAACAACAACAAC	TGACGAGGGGAAGAAGAAGTAGTAGC
WOR2	GCCCTCATCACATTTACGCC	AAACACGTTGGTTTCCGCTC
STP4	TTCGTCGAATTCACCCACACCTTC	AGGAAGCCGGTCTATCGTTACTCG
ADH5	CACGAAATTGCTGGGACTGTTGC	CCGACACAAGCGACACGATCTC
IFD6	TGGTGTCTTGTGTCGTCCATTCG	GCTTCACGAGGTTCACCCAATCC
CSH1	ACCGTGAGGACGAGAGAGAA	GACACAAAACACCACCACCG
GSC1	CAGCTTGGTCTGCTGATCCT	CCTGAGCTGGTGACATACGG
ALG11	TTCCGTCCTGAAAAGCGTCA	TCAGCCGTTCGACAAGATCC
MNN4	GCAATCGTCAAAACCAGTGGA	AAGAATCGTGATGGGCGGTT
ROB1	TGAAGCCTCGTTGGCGAAT	AAGGTGGACGTGAAAGTTTGC
BRG1	TCGCCACATTGCCTCCTTTGATG	GTGTTGCTGTTGTTGTTGTTGTTGTTG
PES1	TGCCTGCAACAGACAAGGTT	TGTCCCTTAACAGTGGCTTGG
SET3	CCATCTCCCACACCAGCATT	CAGGAACCACTTGAGCCCTT

Table S3 RNA-seq data summary

Sample	Total Raw Reads (M)	Total Clean Reads (M)	Total Clean Bases(Gb)	Clean Reads Q20(%)	Clean Reads Q30(%)	Clean Reads Ratio(%)
AMP-17(a)	47.33	42.09	6.31	96.84	92.2	88.93
	47.33	42.41	6.36	96.97	92.55	89.61
	47.33	42	6.3	96.9	92.38	88.76
Control(a)	47.33	42.91	6.44	96.84	92.25	90.68
	49.08	42.45	6.37	96.87	92.34	86.48
	47.33	42.65	6.4	96.83	92.24	90.12
	45.57	44.43	6.66	97.35	93.15	97.49
AMP-17(b)	47.33	43.3	6.49	97	92.68	91.49
	43.82	42.77	6.42	97.17	92.88	97.61
	43.82	42.85	6.43	97.28	93.09	97.79
Control(b)	43.82	42.88	6.43	97.16	92.85	97.85
	43.82	42.83	6.42	97.2	92.91	97.74

(a) Samples are collected in early biofilm stage, cells (1×10^6 CFU/mL) co-incubated with or without 32 μg/mL AMP-17 at 37°C for 12h.

(b) Samples are collected in mature biofilm, cells (1×10^6 CFU/mL) incubated at 37°C for 12h to grow mature, followed co-incubated with or without 128 μg/mL AMP-17 for 12h.
